# Effects of different environmental intervention durations on the intestinal mucosal barrier and the brain-gut axis in rats with colorectal cancer

**DOI:** 10.1038/s41598-022-24861-8

**Published:** 2022-11-28

**Authors:** Liu Dun, Chen Mei-Jing, Huang Si-Ting, Yu Xin-Yuan, Wu Yu-Xuan

**Affiliations:** The School of Nursing, Fujian Medical University, No. 1 Xuefu North Road, Minhou County, Fuzhou, 350122 Fujian China

**Keywords:** Cancer, Health care, Medical research, Oncology

## Abstract

An enriched environment (EE) is a promising strategy for protecting the intestinal mucosal barrier and regulating the brain-gut axis, but the optimal EE intervention duration is unknown. Here, different EE intervention durations were applied to assess the optimal intervention duration in rats with colorectal cancer. We used a rat model of 1, 2-dimethylhydrazine-induced colorectal cancer. The rats were housed in an EE for 0, 2, 4, 8 weeks and 8-week blank group. The intestinal mucosa and serum TNF-α, IL-6, IL-10, ATP, CRF, and occludin levels and bacterial translocation (BT) were measured, and the intestinal mucosa morphology was evaluated. In 8 weeks, the effect of tumor on intestinal mucosal barrier was not obvious and the EE had a greater impact on it. Eight weeks of EE was more beneficial to the intestinal mucosal mechanical barrier than 2 or 4 weeks of intervention. A significant difference in BT was found between the 4- and 8-week groups. Overall, the analysis of inflammatory factor regulation revealed that the two blank groups exhibited the worst effect, and the intervention effect at 8 weeks was better than that at 2 and 4 weeks. CRF at 4 weeks was higher than that at 8-week blank group. The effect of 8-week intervention duration on the intestinal mucosal barrier was generally better than that of 2- and 4-week durations and intervention within 4 weeks can help to stabilize and promote the secretion of brain gut peptide, but the effect of different intervention durations on the brain-gut peptide levels was not obvious. In the future, we can further explore the molecular biological mechanism of the effect of different EE intervention durations on the intestinal mucosal barrier and analyze the effect of an EE on other brain-gut peptides.

## Introduction

An enriched environment (EE) is a paradigm in which animals are introduced to novel, complex, and stimulating surroundings that promote structural changes in the brain and enhance learning and memory performance in rodents^1^. In an EE, mice or rats are placed in larger cages containing multiple physical and social stimuli, which allow the animals to explore, exercise, and interact with each other^2^. Recent studies have investigated the role of EE in cancer treatment and prognosis^2–5^. Several studies have indicated that EE can regulate the intestinal microenvironment and enhance intestinal immunity^6–11^, thereby protecting the intestinal mucosal barrier. According to research on the role of an EE on the intestinal mucosal barrier and brain gut axis, the body’s exposure to stressors will affect the intestinal microbial environment, including Lactobacillus. Intestinal microorganisms are an important part of the intestinal mucosal biological barrier and are closely related to intestinal mucosal barrier function^12,13^. In addition, stressors can also affect the brain gut balance, and its mechanism is mainly to overexpress CRF, the main mediator of central involvement in the stress response, to regulate gastrointestinal motility, secretion and sensation through the HPA axis. EE is an effective method for relieving pressure. Therefore, an EE can improve the intestinal microenvironment by alleviating the stimulus of stressors, protect intestinal mucosal barrier function and maintain the brain-gut balance^14^. In addition, some studies have shown that proper exercise can improve the lifestyle of rats, thereby regulating brain-gut peptides and reducing the inflammatory reaction of serum and intestinal mucosa^15,16^. Therefore, proper exercise can affect the gastrointestinal mucosa and gastrointestinal function of colorectal cancer (CRC) by regulating the brain-gut axis. A study on the relationship between an EE and exercise showed that objects of different colors and shapes, toys and cognitive stimuli can enhance the exercise level of rats^17^. Therefore, rats living in an EE can protect the colonic immune barrier mucosa of rats through active and appropriate exercise.

Previous studies have also shown that a combination of three factors, namely, an enlarged space, social interaction and cognitive stimulation, plays a role in improving the levels of intestinal ghrelin, serum IL-6, intestinal mucosal TNF-α and IL-6, and hypothalamic corticotrophin-releasing factor (CRF)^18,19^. The effect of combining social interaction and cognitive stimulation on the ghrelin levels in the hypothalamus, the TNF-α levels in serum, the IL-10 levels in serum and the intestinal mucosa is better than the effect of the combination of the three factors. However, the combination of the three factors is better than the combination of social interaction and cognitive stimulation in improving the muscle layer thickness and occludin levels. Among the univariate factors, cognitive stimuli improve the TNF-α levels in serum and the IL-10 levels in the intestinal mucosa more than the other two factors^18^. In addition, an EE can regulate the expression of brain-gut peptides^20–23^ and thereby regulates gastrointestinal function. Therefore, an EE is beneficial for protecting the intestinal mucosa.

However, in the above studies, the EE intervention duration ranged from 2 to 18 weeks. The optimal duration of EE intervention is not well known. Therefore, in this study, we used different durations of EE intervention in CRC rats to analyze the optimal EE intervention duration.

## Materials and methods

### Animals

All procedures were approved by the Laboratory of Animal Welfare and Ethics Committee of Fujian Medical University (certificate number 2021-98) and were performed in accordance with the National Institutes of Health guidelines for the treatment of animals. According to the design of a previous study, at least 8–15 rats were included in each group in an EE^3^. Therefore, we selected 12 rats for each group, and a total of 60 rats were included in this study. We obtained 60 4-week-old male Sprague–Dawley rats weighing approximately 150–160 g from the Animal Experimental Center of Fujian Medical University. Prior to the experiments, all the rats were fed adaptively based on the criteria for 2 weeks: the rats were housed in ventilated cages containing four to five rats and soft shavings in an air-conditioned room (22 ± 1 °C, 50–60% humidity, 12-h light–dark cycle), and the rats were fed ad libitum (20–25% protein, 5–10% fat, and 3–5% crude fiber diet). The food was prepared and mixed according to the guidelines set by the Association of Analytical Communities. Rats that did not exhibit loss of agility or loss of teeth were deemed fit for the investigation because rats with a poor mental state and a loss of agility in their activities will be unable to accept an EE intervention and because rats that cannot eat normally due to tooth loss will affect the results from the evaluation of the intestinal mucosa.

### Generation of 1,2-dimethylhydrazine-induced tumors

Some studies have shown that 1,2-dimethylhydrazine (DMH) has the advantages of a high cancer induction rate, a similar tumor nature and inducing progression to human CRC^24,25^. Therefore, DMH was used to induce CRC in rats. Starting at 6 weeks of age, the rats were subcutaneously injected with DMH (Sigma Chemical Co., USA). A solution containing 2% DMH in normal saline was prepared, and the pH was adjusted to approximately 6.5 with NaOH. According to the weight of the rats, a subcutaneous injection of 20 mg/kg was administered once per week for 21 weeks. DMH is highly carcinogenic and has high organ specificity. DMH has been used in many studies to model CRC in rats^26^.

### Animal housing procedures

Twenty-one weeks after DMH injection, all the rats were examined by ultrasonography (Esaote, MyLab Class C, Italy; probe frequency, 18–22 MHz). Tumor formation occurred in all 48 rats, and these animals were then divided into five groups of 12 rats each based on stratified randomization according to their weights. All the rats developed multiple tumors in the gut. The first group was the blank group of 0 week. The EE intervention durations of the second, third, fourth and fifth groups were 2, 4, 8 weeks and 8-week blank, respectively. Previous studies have shown an elevated level of prostaglandin E2 in the colonic mucosa of patients with CRC^27^. Prostaglandin E2 is associated with malignant tumor progression and intestinal barrier function^28^. A higher BMI is associated with a higher level of prostaglandin E2^29^. Therefore, in this study, weight was used as a baseline measure of tumor progression and bowel function in cancer.

For the EE conditions, large cages (109 × 79 × 41 cm) containing 12 rats were used. An EE was established as described in the relevant literature^17,30–32^. Stimulatory objects were placed in the cages. The number of stimulatory objects was approximately 1–2 per rat, and the objects included huts made of wood, walking wheels made of plastic with a diameter of 21 cm, transparent labyrinth tunnels made of acrylic with a diameter of 13 cm and various wooden toys. All the objects were harmless to the rats. Items that had been destroyed by the rats were replaced periodically. In addition, the positions of the stimuli, water, and food in the cages were changed twice weekly.

The rats in the control group of 0 week were killed on the same day after ultrasonic examination. The rats in the second, third and fourth groups were placed into the EE for feeding. The rats of 8 weeks blank group were housed in three standard cages containing four rats each without any stimuli. The rats in the 2-week group were killed after being fed in the EE for 2 weeks. The rats in the 4-week group were killed after 4 weeks in the EE. The rats in the 8-week group and 8-week blank group were killed after being fed in the EE for 8 weeks.

### Measurement of the TNF-α, IL-6, IL-10 and CRF levels

Rat intestines (approximately 400 mg of tissue per sample) were clipped and washed with saline. The samples were then cut into slices, homogenized using a Dounce homogenizer (WHEATON, USA), and centrifuged at 4 °C and 10,000 rpm for 30 min. The supernatants from each fraction were collected and stored at − 80 °C. For serum samples, blood was drawn from the inner canthus vein. Whole blood was incubated at 4 °C for 24 h and then centrifuged at 10,000 rpm for 10 min. Serum was isolated from whole blood using a liquid transfer gun and stored at − 80 °C. All the samples were then thawed, and the levels of TNF-α, IL-6, IL-10 and CRF were determined by enzyme-linked immunosorbent assays (ELISAs) according to the manufacturer’s instructions.

### Detection of bacterial translocation

Mesenteric lymph nodes, livers, and spleens were collected under sterile conditions. One milliliter of cold saline was added to each specimen. The samples were then ground using a mortar and pestle, and 0.5 ml of each sample was incubated with medium containing eosin methylene blue agar at 37 °C for 48 h. The rosin acid contained in the medium inhibited the growth of gram-positive bacteria but had no inhibitory effect on the growth of gram-negative bacteria. Escherichia coli is a gram-negative bacterium that is capable of lactose decomposition and forms blue colonies on this medium, and the size of a single colony was approximately 2 mm^33^. The number of bacterial colonies was counted, and the number of CFUs per gram of tissue (CFUs/g) was calculated. In this study, culture plates harboring more than five colonies indicated a positive result^34^.

### Intestinal mucosal morphology

The rat intestines were isolated, fixed with 10% formaldehyde, dehydrated, and embedded in paraffin, and 5-μm-thick sections were then cut, dewaxed with xylene, hydrated with an alcohol gradient, and stained with hematoxylin for 1 min. The samples were then washed with phosphate-buffered saline (PBS) and stained with eosin for 15 s before being rapidly dehydrated with an alcohol gradient. The sections were treated with xylene, mounted in neutral gum, and viewed with a light microscope. The hematoxylin and eosin (HE) staining results were evaluated using Image-Pro Plus 6.0, and the groups were compared.

### Immunohistochemical detection of occludin

The rat intestines were fixed in 10% formalin for 24 h and embedded in paraffin, and 5-μm thick sections were cut, mounted on slides, and incubated with an anti-occludin antibody (1:120, Thermo, USA) for 2 h at 37 °C. The slides were then washed three times with PBS and incubated with a goat anti-rabbit secondary antibody (Maixin Biological Technology Development Co., Ltd., Fuzhou, China) for 30 min. The slides were washed three times with PBS and developed using diaminobenzidine color development solution (Fuzhou Maixin Biotechnology Development Co., Ltd.) for 5 min. The slides were then stained with hematoxylin for 1 min, washed with PBS, dehydrated with an alcohol gradient, treated with xylene, mounted with neutral gum, and viewed with a light microscope (Nikon SMZ645, Japan). The immunohistochemical staining results were evaluated using Image-Pro Plus 6.0, and between-group comparisons were conducted. The investigator who analyzed all the immunohistochemically stained slides was blind to the group allocation of each sample. The expression levels of occludin were analyzed as described in the relevant literature^35^.

### Statistical analysis

After each rat was cultured, 3 parts were collected for the measurement of BT, and the parts showed positive bacterial culture. We thus used the ratio of the number of positive sites to the number of bacterial cultures to measure the BT. The chi square test was used to test the difference in BT between different groups. The other indices are presented as the means ± standard deviations. The differences between groups were assessed by one-factor analysis of variance (ANOVA) with a post hoc Bonferroni pairwise comparison, and the degrees of freedom were 3. The weight differences were assessed by one-way repeated-measures ANOVA. For nonnormally distributed data, a Kruskal–Wallis test followed by a post hoc Mann–Whitney U test was used for pairwise comparisons. P < 0.05 was considered to indicate significance. An adjusted significance level of P < 0.01 was used for post hoc pairwise comparisons. All statistical analyses were performed using SPSS 24.0 statistics (SPSS Inc., Chicago, IL, USA).

## Results

After the intervention, 4 rats in the 8-week group and 8-week blank group died, respectively, and all the rats in the other groups survived. Therefore, 8 rats in the 8-week group and 8-week blank group, and 12 rats in the other groups survived after the intervention. According to the principle of rat tail suspension test (TST), we grabbed the rat tail to make the rat present an inverted position. If the rats are struggling and trying to escape with strong force, such as running forward, kicking, trying to raise their head, standing upright, it indicates that the rats have good activity and mental state^36^. The result showed that the activity, mental state, fur and teeth of all the rats in the four groups were normal, and no mental depression or fur or tooth loss was detected.

### Microscopic morphology of the intestinal mucosa

Overall, the 8 weeks blank group exhibited the worst effect and 0-week blank group exhibited the second worst effect. The effect of the 8-week intervention time was better than that of the 2-week and 4-week intervention times. Compared with the intervention effect at 2 and 4 weeks, the effect at 2 weeks was better than that at 4 weeks in terms of villus width and muscle layer thickness. In terms of villus length, the 4-week intervention was better than the 2-week intervention.

### Villus length

**Figure 1:**
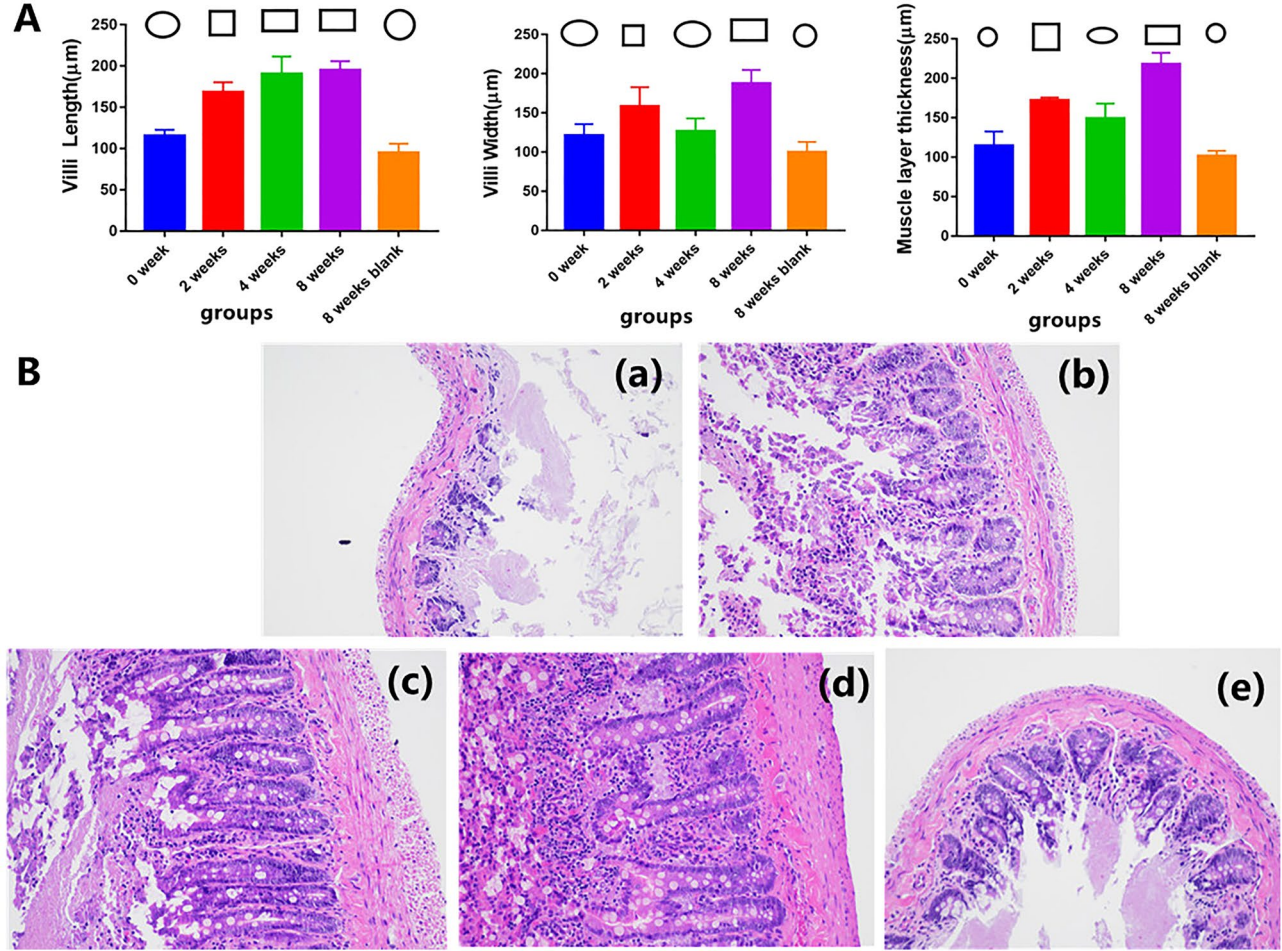
Microscopic morphology of rats with CRC in the different intervention time groups. (**A**) (Circle) The lowest level group compared with the other groups. P ≥ 0.05 compared between each other with the same symbol. P < 0.05 compared between each other with the different symbol. (**B**) Microscopic morphology of rats with CRC in the different intervention time groups. (**a**) 0-week group. (**b**) 2-weeks group. (**c**) 4-weeks group. (**d**) 8-weeks group. (**e**)8 weeks blank group.

Samples were collected from the rat intestines, and pathology methods were used for analysis. A significant effect on the small intestinal villus length was found among the different groups (F = 103.727, P < 0.01). The small intestinal villus length did not significantly differ between the 4- and 8-week groups (P = 0.448). Significant differences were detected among the other groups (Fig. 1).

### Villus width

A significant effect on the small intestinal villus width was found among the different groups (F = 31.639, P < 0.01). The small intestinal villus width did not significantly differ between the blank and 4-week groups (P = 0.455). Significant differences were detected among the other groups (Fig. 1).

### Muscle layer thickness

A significant effect on the small intestinal muscle layer thickness was detected among the different groups (F = 95.122, P < 0.01). No significant difference was found between the 0- and 8-week blank groups (P = 0.052), but significant differences were detected among the other groups (Fig. 1).

### Immunohistochemical detection of occludin

**Figure 2:**
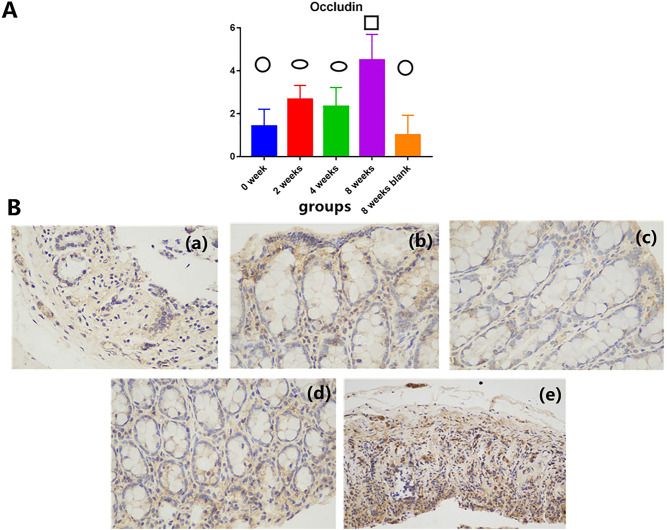
Effect of different environmental intervention durations on the intestinal levels of occludin in rats injected with DMH. (**A**) (Circle) The lowest level group compared with the other groups. P ≥ 0.05 compared between each other with the same symbol. P < 0.05 compared between each other with the different symbol. (**B**) Intestinal epithelial occludin expression of the four groups. (**a**) 0-week group. (**b**) 2-weeks group. (**c**) 4-weeks group. (**d**) 8-weeks group. (**e**) 8 weeks blank group.

Overall, a longer EE intervention duration is more beneficial to the growth of occludin. A significant effect on intestinal occludin was found among the different groups (F = 20.483, P < 0.01). The intestinal occludin staining did not significantly differ between the 2- and 4-week groups (P = 0.357) and between the 0- and 8-week blank groups (P = 0.304). Significant differences were detected among the other groups (Table 1, Fig. 2).

**Table 1:** Effect of different environmental intervention durations on intestinal levels of occludin in rats injected with DMH.

Entry	Group	$\overline{x}$ ±s	*F*	*P*	
Occludin	2.327 ± 1.396	0 week	1.417 ± 0.793^c^	20.483	< 0.01
		2 weeks	2.667 ± 0.651^b^		
		4 weeks	2.333 ± 0.888^b^		
		8 weeks	4.500 ± 1.195^a^		
		8 weeks blank	1.000 ± 0.926^c^		

There is no significant difference in the right superscript with same letter between the two groups. There is significant difference in the right superscript with letter differences.

### Serum and intestinal mucosa levels of TNF-α, IL-6, and IL-10

Serum and intestinal mucosa samples were collected, and ELISAs were used for detection. Overall, in terms of inflammatory factor regulation, the two blank groups exhibited the worst effect, and the intervention effect at 8 weeks was better than that at 2 and 4 weeks. The intervention effect was compared between 2 and 4 weeks. The analysis of the regulation of IL-10 and TNF-α revealed that the intervention effect at 2 weeks was better than that at 4 weeks. In terms of IL-6 regulation, the effect of the 4-week intervention duration was better than that of the 2-week intervention duration.

### Serum levels of IL-10, IL-6 and TNF-α

**Figure 3:**
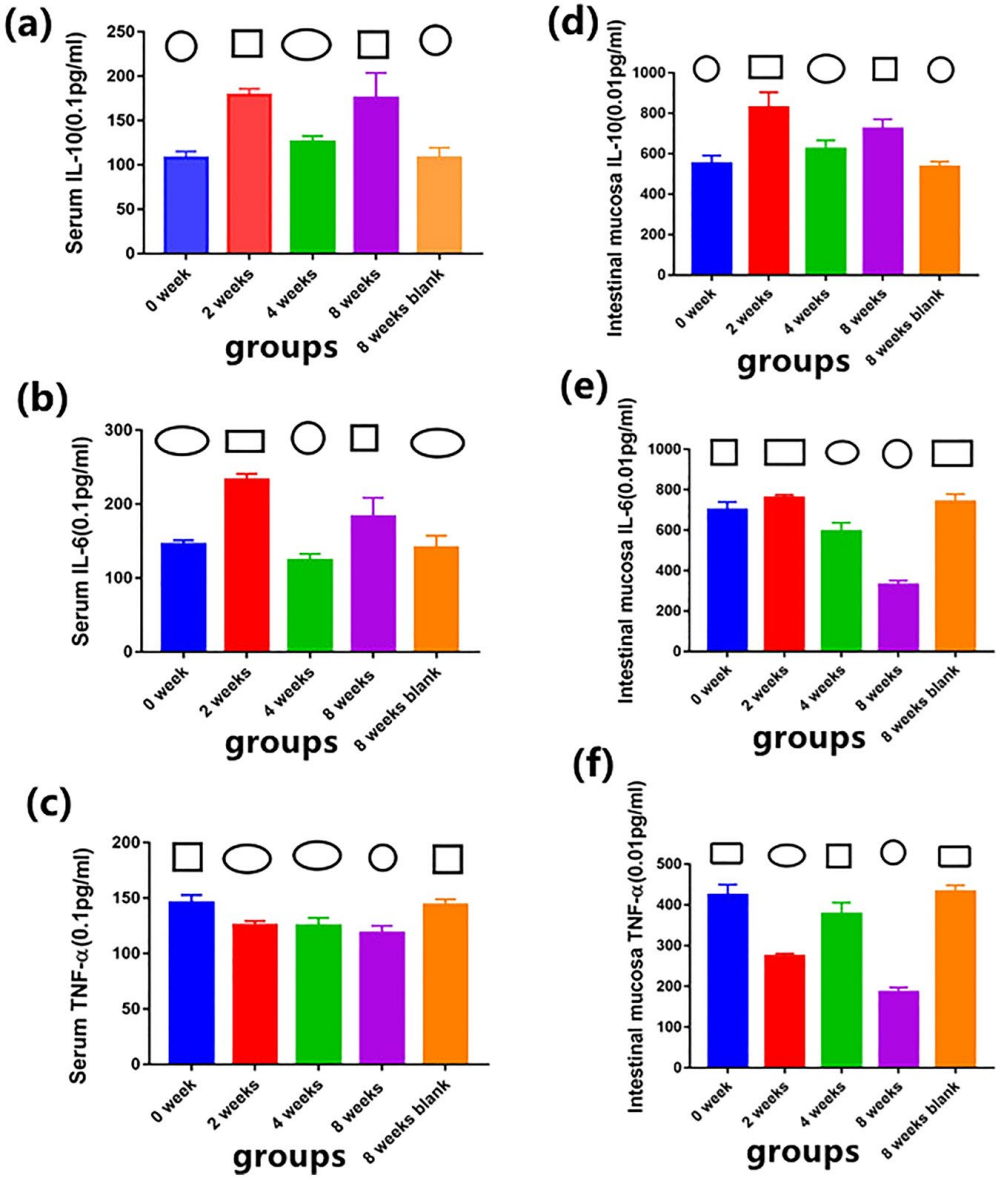
Serum and intestinal mucosal cytokine levels in rats with CRC. (Circle) The lowest level group compared with the other groups. (Square) The highest level group compared with the other groups. P ≥ 0.05 compared between each other with the same symbol. P < 0.05 compared between each other with the different symbol. (**a**) Serum IL-10 level in five groups of rats with colorectal cancer. (**b**) Serum IL-6 level in five groups of rats with colorectal cancer. (**c**) Serum TNF-ɑ level in five groups of rats with colorectal cancer. (**d**) Intestinal mucosal IL-10 level in five groups of rats with colorectal cancer. (**e**) Intestinal mucosal IL-6 level in five groups of rats with colorectal cancer. (**f**) Intestinal mucosal TNF-ɑ level in five groups of rats with colorectal cancer.

A significant effect on the serum levels of IL-10 was found among the different groups (F = 72.301, P < 0.01). The serum levels of IL-10 did not significantly differ between the 2- and 8-week groups (P = 0.563) or between the 0- and 8-week blank groups (P = 0.950). Differences were detected among the other groups (Table 2, Fig. 3).

**Table 2:** Serum and intestinal mucosal cytokine levels in rats with CRC (mean number ± standard deviation, $\overline{x}$  ± s, 0.1 pg/ml).

Groups	Serum cytokine IL-10	Serum cytokine IL-6	Serum cytokine TNF-α	Intestinal mucosal IL-10	Intestinal mucosal IL-6	Intestinal mucosal TNF-α
0 week	108.11 ± 7.16^c^	145.47 ± 5.73^c^	145.57 ± 7.40^a^	550.22 ± 41.17^d^	700.11 ± 39.84^b^	424.10 ± 25.52^a^
2 weeks	178.91 ± 7.13^a^	232.66 ± 8.39^a^	125.24 ± 4.03^b^	828.06 ± 75.28^a^	759.25 ± 15.3^a^	273.62 ± 7.19^c^
4 weeks	125.77 ± 6.79^b^	124.24 ± 8.57^d^	124.96 ± 7.26^b^	622.20 ± 44.07^c^	594.15 ± 42.56^c^	376.78 ± 28.87^b^
8 weeks	175.37 ± 28.58^a^	182.80 ± 25.83^b^	118.17 ± 6.86^c^	722.18 ± 48.44^b^	329.04 ± 21.75^d^	185.21 ± 12.18^d^
8 weeks blank	107.73 ± 11.67^c^	140.86 ± 16.63^c^	143.76 ± 5.21^a^	533.40 ± 27.88^d^	739.58 ± 39.12^a^	431.85 ± 16.46^a^
*F*	72.301	119.037	37.380	62.205	237.643	244.469
*P*	< 0.01	< 0.01	< 0.01	< 0.01	< 0.01	< 0.01

There is no significant difference in the right superscript with same letter between the two groups. There is significant difference in the right superscript with letter differences.

A significant effect on the serum levels of IL-6 was detected among the different groups (F = 119.037, P < 0.01). The serum levels of IL-6 did not significantly differ between the 0- and 8-week blank groups (P = 0.458). Differences were detected among the other groups (Table 2, Fig. 3).

A significant effect on the serum levels of TNF-α was detected among the different groups (F = 37.380, P < 0.01). The serum levels of TNF-α did not significantly differ between the 2- and 4-week groups (P = 0.914) or the 0- and 8-week blank groups (P = 0.534). Differences were detected among the other groups (Table 2, Fig. 3).

### Intestinal mucosa levels of IL-10, IL-6 and TNF-α

A significant effect on the intestinal mucosa levels of IL-10 was found among the different groups (F = 62.205, P < 0.01). The intestinal mucosa levels of IL-6 did not significantly differ between the 0- and 8-week blank groups (P = 0.477). The intestinal mucosa levels of IL-10 did significantly differ among the other groups (Table 2, Fig. 3).

A significant effect on the intestinal mucosa levels of IL-6 was detected among the different groups (F = 237.643, P < 0.01). The intestinal mucosa levels of IL-6 did not significantly differ between the 2-week group and 8-week blank group (P = 0.210). The intestinal mucosa levels of IL-6 showed significant differences among the other groups (Table 2, Fig. 3).

A significant effect on the intestinal mucosa levels of TNF-α was found among the different groups (F = 244.469, P < 0.01). The intestinal mucosa levels of TNF-α did not significantly differ between the 0- and 8-week blank groups (P = 0.413). The intestinal mucosa levels of TNF-α were significantly different among the other groups (Table 2, Fig. 3).

### Weight

Before the intervention, the weights of the rats did not significantly differ among the groups. However, after the intervention, the body weight of the rats in 0-week blank, 2 weeks, 4 weeks, 8 weeks and 8-week blank groups were 580.67 ± 69.86, 580.67 ± 65.68, 570.17 ± 63.61, 500.50 ± 120.71 and 549.63 ± 127.16, respectively. The weight of the rats in different group was still not significantly different (F = 1.322, P = 0.276).

### BT ratios of the three groups

BT occurred in 24 out of 36 tissues in the blank group, 20 out of 36 tissues in the 2-week group, 23 out of 36 tissues in the 4-week group, 8 out of 24 tissues in the 8-week group and 15 out of 24 tissues in the 8-week blank group. A significant effect on BT was found between the 0-week blank and 8-week groups (P < 0.05). No significant differences were found among the other groups. The results indicated that the effect of the 8-week intervention was better than those observed in the other groups, but no significant differences in BT were found among the other groups.

### Serum CRF levels in rats with CRC

Serum samples were collected, and an ELISA was used for detection of the CRF levels. A significant effect on the serum CRF levels was found between the 4-week and 8-week blank groups (P < 0.05). No significant effect on the serum levels of CRF was found among the other different groups (F = 1.072, P = 0.381) (Table 3).

**Table 3:** Effect of different environmental intervention durations on the serum CRF levels of rats injected with DMH after intervention.

Entry	Group	$\overline{x}$± s	*F*	*P*	
Serum CRF	175.93 ± 47.54	0 week	177.20 ± 52.32^ab^	1.072	0.381
		2 weeks	177.95 ± 39.93^ab^		
		4 weeks	191.30 ± 66.14^a^		
		8 weeks	176.90 ± 33.16^ab^		
		8 weeks blank	146.93 ± 17.64^b^		

There is no significant difference in the right superscript with one same letter between the two groups. There is significant difference in the right superscript with letter differences.

### Ethical approval and consent to participate

Male specific pathogen-free (SPF) Sprague‒Dawley (SD) rats were purchased from the Animal Experiment Center of Fujian Medical University. The rats were purchased under license number SCXK (Fujian) 2016-0002. After purchase, the rats were reared at the Animal Experiment Center of Fujian Medical University under license number SYXK (Fujian) 2016-0006. All procedures involving animals were performed in accordance with the ethical standards of the institution or practice at which the studies were conducted (Fujian Medical University, 2021-98). Our manuscript confirms that the study has been reported in accordance with ARRIVE guidelines.

## Discussion

Some studies have shown that an EE not only influences the brain structure and function^37^ but also significantly inhibits tumor growth in colon cancer^5^ .A previous study on EE intervention for 8 weeks in rats with CRC also showed that an EE could increase brain-gut peptide expression (especially ghrelin secretion) and enhance intestinal mucosal immunological functions, thus ameliorating intestinal dysfunction and maintaining the integrity of the intestinal mucosal barrier. However, the best and most effective intervention durations remain unknown. Therefore, this study used different intervention durations for the treatment of CRC rats to analyze the optimal EE intervention duration for rats with CRC to lay a foundation for studying nondrug interventions for clinical cancer patients. However, in this study, all rats formed multiple tumors in the intestine. Therefore, it is impossible to judge whether the number of tumors decreased. Which intervention duration is more beneficial for reducing tumors remains to be further studied.

### Intestinal mucosal mechanical barrier

Intestinal epithelial cells and tight junctions (TJs) between intestinal epithelial cells form the structural basis of the intestinal mucosal mechanical barrier. TJ barrier function can be affected by changes in the distribution of specific TJ proteins and/or their expression levels. The intestinal epithelial transmembrane binding protein occludin is a transmembrane protein and one of the primary TJ proteins among the zonula occludin proteins^38–40^. Therefore, the occludin levels and the length, thickness, and muscular thickness of intestinal epithelial villi can affect the intestinal mucosal mechanical barrier to a certain extent.

The results of this study show that the tumor had a depletion effect on intestinal mucosal villi within 8 weeks. However, enriched environment can repair intestinal mucosa. The effect of the 8-week EE intervention duration is generally better than that of the 2- and 4-week intervention durations. The intervention effect at 2 and 4 weeks was better than that observed in the blank groups. However, whether the 2- or 4-week intervention time is better remains unclear. Therefore, an EE is helpful for maintaining the intestinal mechanical barrier, and the effect of longer intervention times is better than that of shorter intervention times. The effect obtained with less than 4 weeks of intervention may not be superior.

### Intestinal mucosal immune barrier

Cytokines are the major regulators of mucosal immunity and are important for the intestinal immune defense response^21^. Cancer patients generally exhibit changes in cytokine levels, which greatly affect the metabolism and immunity in the body. There are two primary types of cytokines: (1) those that promote the inflammatory reaction, such as TNF-α, IL-1, and IL-6, and (2) those involved in the suppression of inflammatory response factors, such as IL-4 and IL-10^41^.

Our results showed that tumor had no significant effect on inflammatory factors except for intestinal mucosa IL-6 within 8 weeks. An EE can adjust the IL-10, IL-6, and TNF-α levels to exert beneficial effects on the body. However, differences were found among the groups with different intervention times. The serum IL-6 levels of the 4-week group were lower than those of the other groups. The serum levels of IL-10 found in the 2- and 8-week groups were higher than those found in the 4-week group, and the levels of serum IL-10 in the three intervention groups were higher than those in the two blank groups. The serum TNF-α level of the 2- and 4-week groups was higher than that of the 8-week group, and the serum TNF-α level of the three intervention groups was lower than that observed in the two blank groups. The intestinal mucosa level of IL-10 found in 2- and 8-week groups were higher than those found in 4-week group, and the levels of intestinal mucosa IL-10 in the three intervention groups were higher than those in the two blank groups. The intestinal mucosa level of IL-6 found in 8-week groups were lower than those found in 2- and 4-week groups. The intestinal mucosa level of IL-6 found in 8-week blank group were higher than those found in 0-week groups. The intestinal mucosa TNF-α level of the 2- and 4-week groups was higher than that of the 8-week group, and the intestinal mucosa TNF-α level of the three intervention groups was lower than that observed in the two blank groups.

Therefore, an EE generally helps to regulate inflammatory factors and thereby protect the intestinal immune barrier. The effect of different intervention durations was better than that observed in the blank groups, and the intervention effect at 8 weeks was slightly better than that at 2 and 4 weeks. However, the effect of different intervention times on the intestinal mucosal immune barrier needs to be further studied with respect to additional inflammatory factors to obtain a clearer conclusion.

### Intestinal mucosal biological barrier

BT refers to the translocation of intestinal bacteria from the intestinal lumen to the mesentery or other organs. Normally, intestinal BT does not occur easily due to intestinal TJs. However, BT in the intestinal tract increases during bacterial pathogenesis or during periods of stress when the mucosal epithelium is damaged. Therefore, BT can be used to evaluate the permeability of the intestinal mucosal barrier^42^. In this study, although a difference in BT was only found between the 0-week blank and 8-week groups, the regulatory effect of the 8-week intervention on BT was generally better than that of the 2- and 4-week interventions. Therefore, after a sufficient EE intervention time, the intervention can regulate the intestinal mucosal biological barrier. These results suggest that adequate cognitive, psychological, sport and other nondrug intervention measures are conducive to improving the intestinal mucosal biological barrier function. However, the specific mechanism needs further study.

### Weight

Due to the presence of multiple tumors, accurately measuring the weight of removed tumors is impossible. The results reveal no significant change in the body weight of rats over 8 weeks of intervention. Therefore, whether the EE is beneficial to the weight maintenance of rats with a long course of disease needs further study. Combined with the survival of rats, the results indicate that the death of rats after 8 weeks of intervention may be due to the consumption of tumors on the body.

### Brain-gut peptide

CRF is the primary mediator that allows the central nervous system to participate in stress responses. Under stress conditions such as disease, CRF can be overexpressed to regulate gastrointestinal motility, secretion and sensation through the hypothalamic‒pituitary‒adrenal (HPA) axis^43^. Regarding the regulation of CRF expression, the tumor can cause the decrease of brain gut peptide secretion within 8 weeks. However, EE intervention, especially within 4 weeks, can stabilize and promote the secretion of brain gut peptide to a certain extent. Thus, the effect of EE intervention on the intestinal mucosa may occur through regulation of the brain-intestine axis. After 4 weeks, the effect of EE on intestinal mucosa may be weakened due to tumor consumption. Therefore, the effects and mechanisms of different intervention durations on the brain-intestine axis need to be further investigated.

## Conclusions

Although tumor may cause slight damage to intestinal mucosal barrier function, enriched environment can protect intestinal mucosal barrier to some extent. The effect of an 8-week environmental intervention duration on the intestinal mucosal barrier was generally better than that of a 2- or 4-week duration and enriched environment within 4 weeks can help to stabilize and promote the secretion of brain gut peptide, but the effect of different environmental intervention durations on the brain-gut peptide levels was not obvious. In the future, we can further explore the molecular biological mechanism underlying the effect of different EE intervention durations on the intestinal mucosal barrier and analyze the effect of an EE on other brain-gut peptides. This study lays a foundation for clinical nondrug intervention research for cancer.

## Data Availability

The datasets used and/or analyzed during the current study available from the corresponding author on reasonable request. Others can replicate and build upon the authors’ published claims. The authors can make materials, data, code, and associated protocols promptly available to readers without undue qualifications. Any restrictions on the availability of materials or information must be disclosed to the editors at the time of submission. Any restrictions must also be disclosed in the submitted manuscript.
